# Extraction and Analysis of Blue Steel Roofs Information Based on CNN Using Gaofen-2 Imageries

**DOI:** 10.3390/s20164655

**Published:** 2020-08-18

**Authors:** Meiwei Sun, Yingbin Deng, Miao Li, Hao Jiang, Haoling Huang, Wenyue Liao, Yangxiaoyue Liu, Ji Yang, Yong Li

**Affiliations:** 1 College of Geographical Science, Harbin Normal University, Harbin 150025, China; meiwei_sun@163.com (M.S.); wenyue_liao@163.com (W.L.); 2 Key Laboratory of Remote Sensing and GIS Application in Guangdong Province, Public Laboratory of Geospatial Information Technology and Application in Guangdong Province, Guangzhou Institute of Geography, Guangzhou 510070, China; yingbin@gdas.ac.cn (Y.D.); haojiang@gdas.ac.cn (H.J.); haoling@163.com (H.H.); lyxy@lreis.ac.cn (Y.L.); yangji@gdas.ac.cn (J.Y.); liyong@gdas.ac.cn (Y.L.); 3 Southern Marine Science and Engineering Guangdong Laboratory (Guangzhou), Guangzhou 511458, China

**Keywords:** CNN, blue steel roofs, GaoFen-2, DeeplabV3+

## Abstract

Blue steel roof is advantageous for its low cost, durability, and ease of installation. It is generally used by industrial areas. The accurate and rapid mapping of blue steel roof is important for the preliminary assessment of inefficient industrial areas and is one of the key elements for quantifying environmental issues like urban heat islands. Here, the DeeplabV3+ semantic segmentation neural network based on GaoFen-2 images was used to analyze the quantity and spatial distribution of blue steel roofs in the Nanhai district, Foshan (including the towns of Shishan, Guicheng, Dali, and Lishui), which is the important manufacturing industry base of China. We found that: (1) the DeeplabV3+ performs well with an overall accuracy of 92%, higher than the maximum likelihood classification; (2) the distribution of blue steel roofs was not even across the whole study area, but they were evenly distributed within the town scale; and (3) strong positive correlation was observed between blue steel roofs area and industrial gross output. These results not only can be used to detect the inefficient industrial areas for regional planning but also provide fundamental data for studies of urban environmental issues.

## 1. Introduction

Blue steel panels have the advantages of being lightweight, easy-to-install, cost-effective, and fireproof [1]. With these economic attributes, blue steel panels have been widely used in roof construction in many inefficient industrial areas (factories and warehouses) and gymnasiums [2]. The distribution of blue steel panels also, directly and indirectly, reflects the urban industrial structure and economic development. For example, the construction of blue steel roofs is largely related to inefficient industrial land [3,4]. In addition, blue steel roofs are also an important part of urban surface areas. Their wide application not only brings convenience to production and life but also negative effects, such as the urban heat island effect. Some researchers have explored the relationship between blue steel roofs and the urban heat environment. For example, some studies have demonstrated a positive correlation between the proportion of blue steel roofs and land surface temperature with an R^2^ of 0.71 [5]. Thus, information on blue steel roofs can provide data to support the study of urban industrial structure and also contribute to the study of urban ecology.

Currently, remote sensing technology is a powerful tool that provides detection and monitoring information for blue steel roofs. These methods include an object-oriented method [6] and spectral index [7] etc. However, within these methods, limitations still exist which influence accuracy. For example, Li [6] analyzed colored steel sheds by an object-oriented model based on Gaofen-1 image and showed that the extraction accuracy in two experimental areas was greater than 88%. The setting of separation scale in image segmentation needed a lot of repeat tests. In addition, the shape of the segmented results could not be perfectly matched the shape of the objects, reducing the extraction accuracy. Guo et al. [7] developed a BSTBI (blue steel tile building index) to extract blue steel roofs using TM (Thematic Mapper) images with the aid of the spectral characteristics of them. The overall accuracy was greater than 85%, while old blue steel tiles covered with dust, along with the lack of homogeneity in meteorological factors, suppressed the performance of indexes and affected the threshold settings associated with the results, thus reducing the accuracy of extraction. Compared with the above methods, deep learning would be a better choice because it has a more powerful and abstract learning ability and higher image recognition accuracy which may improve the weak automation in the object-oriented method. On the other hand, deep learning method can weaken the influence of dust and meteorological factors by increasing the samples of the training dataset.

Hinton et al. [8] proposed the concept of a deep frame neural network, this network showed improved performance and reduced complexity of image segmentation [9]. The deep learning model has been widely applied in geography, medicine, and physics [10,11,12,13,14,15,16,17,18]. For image recognition applications, the most important network structure in the deep learning algorithm is the CNN (Convolutional Neural Network) structure, which has the advantage of enabling computers to automatically extract feature information [19]. Many groups of researchers have begun to use CNN in many applications with impressive performance, such as image classification [20,21], object recognition [22,23], land use [24,25], and semantic segmentation [26,27].

With the increasing demand for practical work in recent years, deep semantic segmentation algorithms have been widely used in remote sensing image processing. The DeeplabV3+ [28] model developed by Google in 2018 is an example of a deep learning algorithm. From the fully convolutional network proposed in 2014 [26] to the DeeplabV3+ in 2018 in the field of image semantic segmentation, the detection effect and performance of these algorithms on public natural scene data sets have increased. Specifically, the Mean Intersection Over Union of the DeeplabV3+ algorithm in the public dataset PASCAL VOC 2012 reached 89%, which is a significant improvement over the previous algorithm [29]. Thus, the use of DeepLabV3+ for remote sensing image segmentation has received increased attention by researchers [30,31,32,33].

For example, Wang and Li [31] used public datasets for model training, applied the DeeplabV3+ network to road network recognition and found that the road extraction accuracy could reach 77.2% at a single scale. In addition, Fang [33] generated a dataset based on Google Earth and also applied the DeepLabV3+ to road network extraction, achieving an accuracy of 86.06%. Liu et al. [30] improved the network in light of the deficiencies of the DeeplabV3+ network, and the accuracy of verification in the high-resolution remote sensing image dataset reached 85%. Tang et al. [34] employed the DeeplabV3+ model and the traditional supervised classification method to extract grassland information simultaneously and found that the DeeplabV3+ extraction accuracy could reach 79.82%, which is higher than the traditional supervised classification method by 5%. Under continuous experiment and verification of a large number of datasets, the segmentation results based on the DeeplabV3+ network had a higher accuracy and a more significant effect.

The Gaofen-2 remote sensing images of the Nanhai District (Lishui, Dali, Shishan, Guicheng) of Foshan, Guangdong Province, China that recorded in 2016 have been used to extract blue steel roofs information based on the DeepLabV3+ deep learning model, followed by a discussion of the patterns of the spatial distribution of blue steel roofs and influencing factors. The findings provide important data for enhancing the ability to identify types of industrial areas with blue steel roofs and could also be used to enhance the construction and management of urban settlements.

## 2. Study Area and Data

### 2.1. Study Area

**Figure 1:**
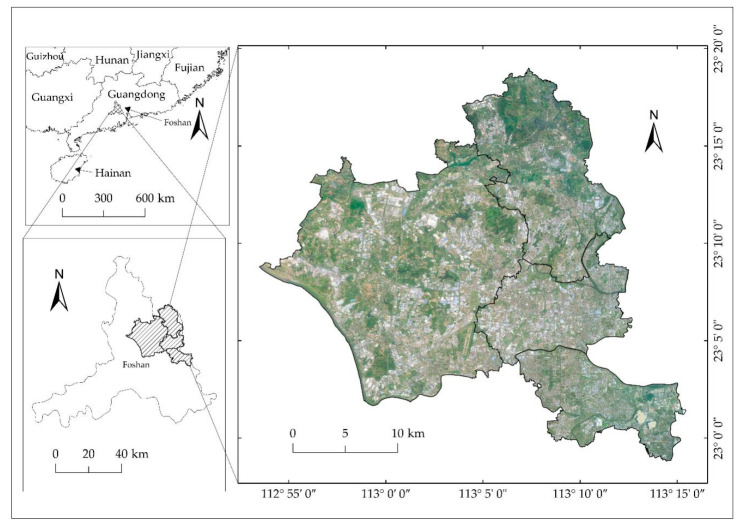
Study area.

The extraction of blue steel roofs data focused on the Nanhai District (Lishui, Dali, Shishan, and Guicheng). The Nanhai District, one of the five administrative districts in Foshan City, Guangdong Province, is a representative example of rural industrialization [35] and an important part of the “Guangfo-Metropolitan Circle”, “Guangfo-Zhao Economic Circle”, and “Zhujiang-Xijiang Economic Belt”. It is located in the hinterland of the Guangdong-Hong Kong-Macao Greater Bay Area, between 22° 48′ 03″–23° 19′ 00″ N and 112° 49′ 55″–113° 15′ 47″ E (Figure 1).

Shishan is dominated by plains and is located in the middle of Nanhai District, Foshan City, Guangdong Province, close to Guangzhou, Hong Kong, and Macao, which is the core area of the economic circle of the Guangzhou-Foshan area in the Pearl River Delta and the kernel park of the Foshan National Hi-Tech Industrial Development Zone. In 2014, it ranked third among the National Comprehensive Strength Top 100 towns and ranked first in Guangdong Province. In 2019, it ranked second among the National Comprehensive Strength Top 1000 towns. Lishui is located in the northeastern part of Nanhai District more than 10 km away from the center of Foshan in the south and was selected as a National Key Town and was among the National Top 100 Towns. In 2019, it was also ranked 10th in the National Comprehensive Strength Top 1000 towns. Dali is located in eastern Nanhai District, north of the Pearl River Delta and adjacent to Guangzhou, which is a coastal area connected to Baiyun District, Liwan District, Guangzhou City in the east and Chancheng District of Foshan City in the south. It is an important link between the two urban centers of Guangzhou and Foshan and was rated as a National Comprehensive Strength Top 1000 town in 2019. Guicheng is located in Nanhai district in the Pearl River Delta, which is the political and economic center of Nanhai District and is also one of the components of Chancheng, Guicheng, and Foshan New City in the central urban area of Foshan. It is also the national base for the jewelry and jade ornaments industry, Guangdong machinery equipment professional town, and strong towns in education and sports in Guangdong Province. According to high-resolution images and Google Earth, blue steel is the main building material in the industrial zone of the study area.

The China Statistical Yearbook shows that the proportion of secondary industry in Foshan ranks first in China. The Nanhai District Statistical Yearbook shows that 5.52 million m^2^ of workshops have been built in Nanhai district from 2012 to 2018 and that the proportion of secondary industry in Nanhai District exceeded 50%, with fluctuations between 50% and 65% from 2005 to 2018. The population and industrial output values of each town in 2016 are shown in Table 1.

### 2.2. Data and Preprocessing

The images used in this study were collected in 2016 which was the first year of the thirteenth Five-Year Plan of China’s national economy. The data source for the remote sensing images was fused using the panchromatic and multispectral images of Gaofen-2. Gaofen-2 includes one 1 m panchromatic and one 4 m multispectral high resolution camera. It has the characteristics of high positioning accuracy, high spatial resolution and fast attitude maneuver. ENVI (The Environment for Visualizing Images) was used for radiometric calibration, atmospheric correction, ortho-rectification, and image fusion. During the preprocessing of remote sensing images, there were four steps: (1) ortho-correction processing of the panchromatic and multispectral imaging of Gaofen-2 images with the Rational Rectification tool based on the Rational Polynomial Coefficient; (2) radiometric calibration, which converts the brightness value of the image into absolute radiation; (3) fast line-of-sight atmospheric analysis of spectral hypercubes in ENVI to conduct an atmospheric correction; and (4) NNDiffuse (nearest-neighbor diffusion) Pan Sharpening to fuse the panchromatic and multispectral images to obtain a 0.8 m high-resolution multispectral fusion image [36].

## 3. Materials and Methods

### 3.1. DeeplabV3+ Architecture

**Figure 2:**
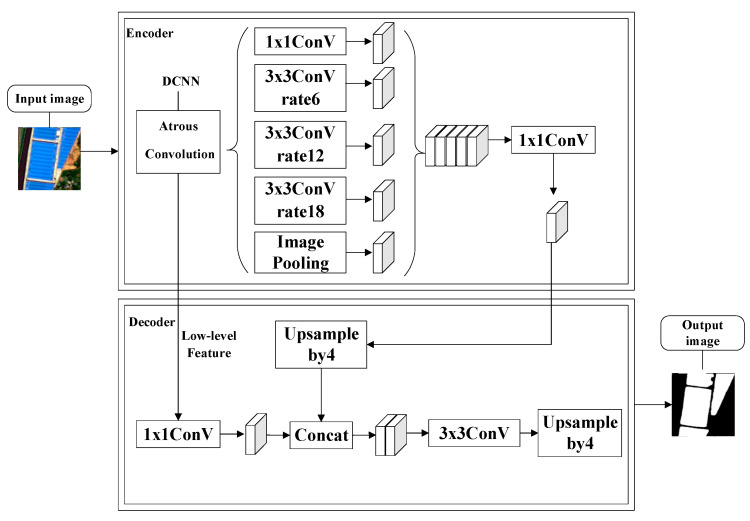
DeepLabV3+ semantic segmentation model [28].

DeeplabV3+ is a semantic segmentation algorithm recently released by Google in 2018, which was developed from DeeplabV1-3 [37,38,39]. The DeeplabV1-3 model has defects, such as slow training speed and low accuracy of target segmentation. DeeplabV1 first involved the Dilated Convolution layer operation, but there was a problem associated with the poor processing capability of multiscale segmentation objects. For this reason, DeeplabV2 was developed with the Atrous Spatial Pyramid Pooling (ASPP) structure based on V1. To compensate for the defects in DeeplabV2, DeeplabV3 changed the ASPP structure to three 3 × 3 convolution operations and a global-average-pooling operation. The DeeplabV3+ network (as shown in Figure 2) provides an encoder-decoder structure based on the DeeplabV3 series of algorithms, which makes DeeplabV3+ produce a faster and more powerful network. The encoder is divided into a deep convolutional neural network and an ASPP layer, and the decoder fuses the low-level features and recovers the feature map [30].

The DeepLabV3+ network combines a deep convolutional neural network (DCNN) and dense conditional random fields, featuring the advantages of DeepLab, Pyramid Scene Parsing Network, and encoder-decoder, representing the highest level in the field of semantic segmentation. First, the fully convolutional deep neural network utilizes convolutional layers to extract the original image features. Second, the ASPP module is used to extract the input features of remote sensing images by multiratio, multieffective domain convolution, and the multiscale context information is encoded by a pool operation. Finally, in the decoding stage, the input image is down-sampled, and the low-level features are fused in the feature map restoration process. The target spatial information is gradually restored to obtain a more accurate target boundary of the remote sensing image [30].

### 3.2. Extraction of Blue Steel Roofs Information

#### 3.2.1. Training Sample Preparation and Model Training

**Figure 3:**
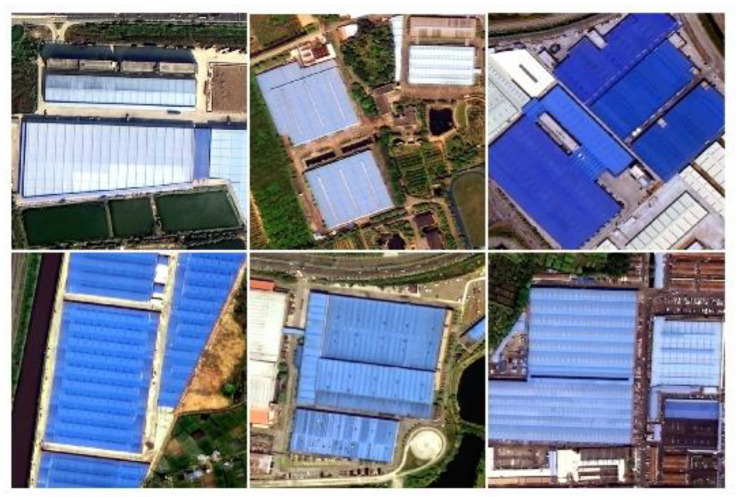
Blue steel roofs sample.

In this study, 8000 samples, created using LabelMe tool, were selected in the Gaofen-2 images for model training. Then, 10 percent of the samples were used as validation while 90 percent were utilized as training samples. These samples consist of roofs with blue dyestuff with no impurities, and most were dark blue (Figure 3).

Training samples were divided into small image sets with sizes of 256 × 256 pixels, and images without marked samples were deleted. Next, images with labeled samples were converted into TFRecord format and applied to train model. The major parameters in the training process are shown in Table 2. Finally, the blue steel roofs in the study area were then inferenced with the trained model.

#### 3.2.2. Evaluation

**Figure 4:**
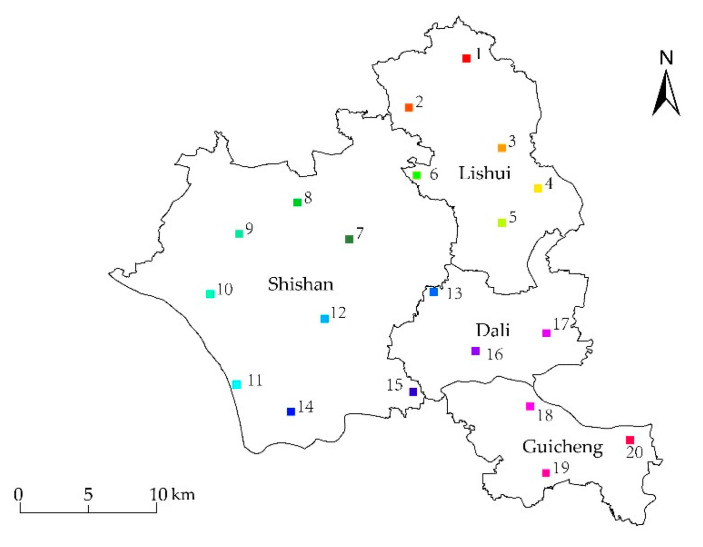
Distribution of the validation samples.

**Figure 5:**
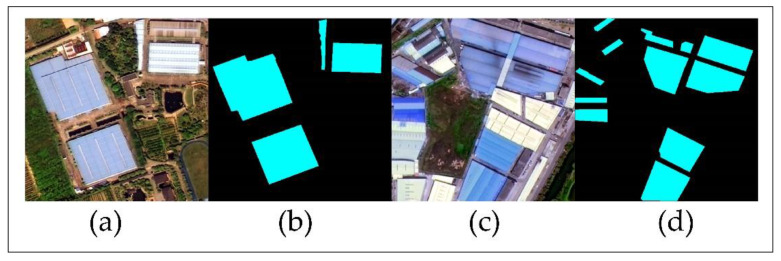
Schematic diagram of digitalized blue steel roofs: (**a**) is the GF-2 image of the sixth region, (**b**) is the digitalized result of the sixth region, (**c**) is the GF-2 image of the tenth region, and (**d**) is the digitalized result of the tenth region.

Since the validation process within DeepLabV3+ model only compares the divided images of training and testing, it cannot verify the whole footprints of the blue steel roofs at the same time. Thus, we also selected another 20 testing samples with 500 × 500 pixels (Figure 4) which could contain the whole footprint of the blue steel roofs building to assess the whole accuracy estimated by DeepLabV3+ model. In addition, testing samples were evenly distributed in the study area based on their according area. Lishui and Shishan contained six and eight verification samples while Guicheng and Dali have three verification samples. Then, the distribution of the blue steel roofs in each verification sample was digitized (Figure 5), and the area of blue steel roofs in each sample was calculated.

Four types of evaluation indexes were used to evaluate the extracted accuracy of blue steel roofs information. The indexes used the confusion matrix, which was generated by the actual area of blue steel roofs in the verification sample and the predicted area of blue steel roofs. True positive (*TP*) indicates the number of pixels correctly classified as blue steel roofs, and false positive (*FP*) indicates the number of pixels misclassified as blue steel roofs. False negative (*FN*) indicates the number of pixels misclassified as background, and true negative (*TN*) indicates the number of pixels correctly classified as background.

The first index is accuracy:

(1) $$Accuracy=\frac{TP+TN}{TP+FP+TN+FN}$$

Generally, accuracy is used to evaluate the accuracy of a classifier relative to the samples overall. It can intuitively reflect the model’s ability to evaluate the whole sample; that is, it can determine the positive as positive and the negative as negative. Accuracy values for the predicted result range between 0 and 1. In this study, Accuracy values are used to indicate the overall accuracy of detection.

The second and third indexes are precision and recall:

(2) $$Precision=\frac{TP}{TP+FP}$$

(3) $$Recall=\frac{TP}{TP+FN}$$

In the study of roof recognition by DCNN, precision and recall can reflect the classifier’s performance. Precision reflects the proportion of real positive samples in the positive cases determined by the classifier, while recall reflects the proportion of positive cases correctly determined to the total positive cases. Precision and recall are generally inversely related; if both are low however, there is a problem with the network.

The fourth index is the *F*1-score:

(4) $$F1-score=\frac{2\cdot Precision\cdot Recall}{Precision+Recall}$$

The *F*1-score is a compromise index, which considers not only the precision of positive samples but also the recall. It is an indicator of comprehensive performance, which is synthetically estimated through the harmonic average. Only when the recall rate and precision rate are high is the *F*1-score high. The *F*1-score can range between 0 to 1, with higher values correspond to higher quality.

### 3.3. Spatial Distribution Analysis of Blue Steel Roofs

#### 3.3.1. Geographic Concentration Index

The geographic concentration index (G) is an important indicator for measuring the degree of agglomeration of research objects. This study was focused on using this index to analyze the spatial distribution of blue steel roofs in the study area. Thus, G was defined as [40]

(5) $$G=100\times \sqrt{\sum_{i=1}^{n}{\left(\left|\frac{x_{i}}{T}\right|\right)}^{2}}$$

where $x_{i}$ is the blue steel roofs area of the ith region; T is the total area of blue steel; and n is the total number of regions. The value of G ranges between 0 and 100. Larger G values correspond to more concentrated distributions. In contrast, smaller G values correspond to more dispersed distributions.

#### 3.3.2. Distribution Homogeneity

Distribution homogeneity (*C*) is an important method for studying the spatial distribution of geographically discrete regions. It can be used to describe or compare the spatial distribution of elements [41,42]. Changes in the regional spatial distribution can be determined by comparing differences in the regional distribution of different research objects:

(6) $$C=1-\frac{-\sum_{i=1}^{N}P_{i}lnP_{i}}{lnN}$$

Here, $P_{i}$ is the proportion of blue steel roofs area of the ith region out of the total blue steel roofs area of the region, N is the total number of regions, and C is the distribution homogeneity. Uniformity values range between 0 and 1, and larger values correspond to more uniform distributions. Following the grading standards that have been used in the analysis of the equity in health resource allocation [43], specifically, the distribution is considered to be uneven when C is under 0.3, generally uniform when C is between 0.3 to 0.6, and uniform when C is above 0.6. This method is used to measure the uniformity of the spatial distribution of blue steel roofs in towns and villages.

#### 3.3.3. Barycenter Model

The idea behind the analytical method of the regional center is to determine the location of the center of the research object in the region and the changes in different years to capture the spatial distribution characteristics of the research object [44]. It is also an important indicator for the study of regional spatial structure. In this study, the mean center is used to represent the average center of the distribution of blue steel roofs in the study area, and the weighted mean center is used to represent the actual distribution center of blue steel roofs in the study area.

The mean center was determined by the following equation:

(7) $$\bar{X}=\frac{\sum_{i=1}^{n}x_{i}}{n}\;\bar{Y}=\frac{\sum_{i=1}^{n}y_{i}}{n}$$

where $\bar{X}$, $\bar{Y}$ are the coordinates of the mean center, $\;x_{i}$, $y_{i}$ are the coordinates of the ith pixel, and n is the total number of pixels.

The weighted mean center was determined by the following equation:

(8) $$\bar{X}=\frac{\sum_{i=1}^{n}x_{i}}{n}\;\bar{Y}=\frac{\sum_{i=1}^{n}y_{i}}{n}$$

where $\bar{X}$, $\bar{Y}$ are the coordinates of the weighted mean center, $x_{i}$, $y_{i}$ are the coordinates of the ith pixel, n is the total number of pixels, and $\omega_{i}$ are the weights of ith pixel [45].

### 3.4. Analysis of Influencing Factors of Blue Steel Roofs Area

The experiment used Pearson correlations to analyze the correlation between the area of blue steel roofs and other influencing factors (GVIOADS, RLF, ICECADS, Population, and GVIO). Pearson correlation coefficients were calculated based on statistical data (Table 1) that reflected the relative strength of each influencing factor. The following formula was used for the calculations:

(9) $$R_{\mathrm{GVIOADS}}=\frac{\sum_{a=1}^{n}\left(X_{a}-{\bar{X}}_{a}\right)\left(Y_{a}-\bar{Y}\right)}{\sqrt{\sum_{a=1}^{n}{\left(X_{a}-{\bar{X}}_{a}\right)}^{2}}\sqrt{\sum_{i=1}^{n}{\left(Y_{a}-\bar{Y}\right)}^{2}}}$$

(10) $$R_{\mathrm{RLF}}=\frac{\sum_{b=1}^{n}\left(X_{b}-{\bar{X}}_{b}\right)\left(Y_{b}-\bar{Y}\right)}{\sqrt{\sum_{b=1}^{n}{\left(X_{b}-{\bar{X}}_{b}\right)}^{2}}\sqrt{\sum_{b=1}^{n}{\left(Y_{b}-\bar{Y}\right)}^{2}}}$$

(11) $$R_{\mathrm{ICECADS}}=\frac{\sum_{c=1}^{n}\left(X_{c}-{\bar{X}}_{c}\right)\left(Y_{c}-\bar{Y}\right)}{\sqrt{\sum_{c=1}^{n}{\left(X_{c}-{\bar{X}}_{c}\right)}^{2}}\sqrt{\sum_{c=1}^{n}{\left(Y_{c}-\bar{Y}\right)}^{2}}}$$

(12) $$R_{P}=\frac{\sum_{d=1}^{n}\left(X_{d}-{\bar{X}}_{d}\right)\left(Y_{d}-\bar{Y}\right)}{\sqrt{\sum_{d=1}^{n}{\left(X_{d}-{\bar{X}}_{d}\right)}^{2}}\sqrt{\sum_{d=1}^{n}{\left(Y_{d}-\bar{Y}\right)}^{2}}}$$

(13) $$R_{\mathrm{GVIO}}=\frac{\sum_{e=1}^{n}\left(X_{e}-{\bar{X}}_{e}\right)\left(Y_{e}-\bar{Y}\right)}{\sqrt{\sum_{e=1}^{n}{\left(X_{e}-{\bar{X}}_{e}\right)}^{2}}\sqrt{\sum_{e=1}^{n}{\left(Y_{e}-\bar{Y}\right)}^{2}}}$$

where $R_{\mathrm{GVIOADS}}$ is the correlation coefficient between the area of blue steel roofs and GVIOADS, $X_{a}$ is the GVIOADS of the ath town, $Y_{a}$ is the area of blue steel roofs of the ath town, and ${\bar{X}}_{a}$ is the mean value of GVIOADS. $R_{\mathrm{RLF}}$ is the correlation coefficient between the area of blue steel roofs and RLF, $X_{b}$ is the RLF of the bth town, $Y_{b}$ is the area of blue steel roofs of the bth town, and ${\bar{X}}_{b}$ is the mean value of RLF. $R_{ICECADS}$ is the correlation coefficient between the area of blue steel roofs and ICECADS, $X_{c}$ is the area of blue steel roofs of the cth town, $Y_{c}$ is the area of blue steel roofs of the cth town, and ${\bar{X}}_{c}$ is the mean value of ICECADS. $R_{P}$ is the correlation coefficient between the area of blue steel roofs and Population, $X_{d}$ is the population of the dth town, $Y_{d}$ is the area of blue steel roofs of the dth town, and ${\bar{X}}_{d}$ is the mean value of Population. $R_{GVIO}$ is the correlation coefficient between the area of blue steel roofs and GVIO, $X_{e}$ is the population of the eth town, $Y_{e}$ is the area of blue steel roofs of the eth town, and ${\bar{X}}_{e}$ is the mean value of GVIO. $\bar{Y}$ is the mean value of the area of total blue steel roofs, and n is the number of samples.

Values of the correlation coefficient range between −1 to 1. Negative values represent negative correlations, and positive values represent positive correlations. Values corresponding to −1 and 1 correspond to perfect correlations and thus linear relationships. The absolute value of the correlation coefficient indicates the degree of correlation between the two variables. Values closer to 1 indicate closer relationships, whereas values closer to 0 indicate weaker relationships. The strength of the correlation expressed by different values of different correlation coefficients is shown in Table 3 and was based on [46].

## 4. Results

### 4.1. Accuracy Evaluation

**Figure 6:**
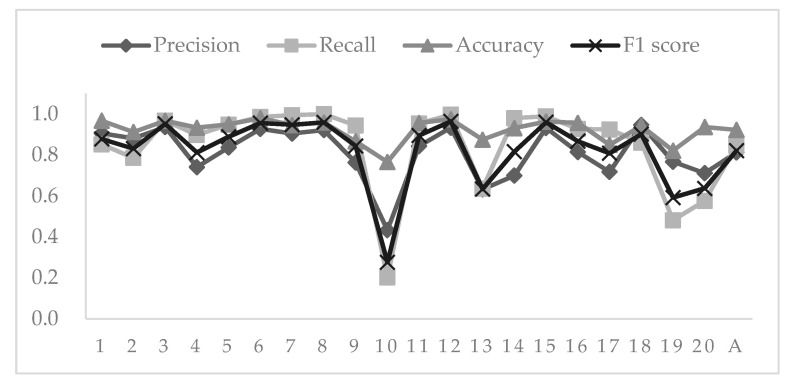
Accuracy assessment based on four indexes. A stands for average, and numbers 1–20 indicate the serial numbers corresponding to the verification samples.

The four accuracy evaluation indexes described above were used to estimate the accuracy of 20 areas for verification. The value of each index ranged between 0 and 1, with larger values corresponding to higher accuracy. The accuracy verification results of the four indicators and the average value of each indicator are shown in Figure 6.

The mean values of precision and recall were 0.81 and 0.84, respectively. The mean value of the F1-score of the 20 samples was 0.82, meaning that the classifier has good performance. This result can also be incarnated in the 800 samples which were used as validation; the accuracy of the DeepLabV3+ model is 70%. However, the mean value of accuracy was 0.92, indicating that a high extraction accuracy was achieved. In the 20 validation samples, there were eight samples with accuracy values higher than 0.9, and only one sample had an accuracy lower than 0.6. Recall values for 12 samples were above 0.9 and below 0.6 for four samples. Overall, there were six samples wherein all four indexes had values above 0.9, and the tenth sample had the lowest values for all four indicators.

### 4.2. Spatial Distribution of Blue Steel Roofs

**Figure 7:**
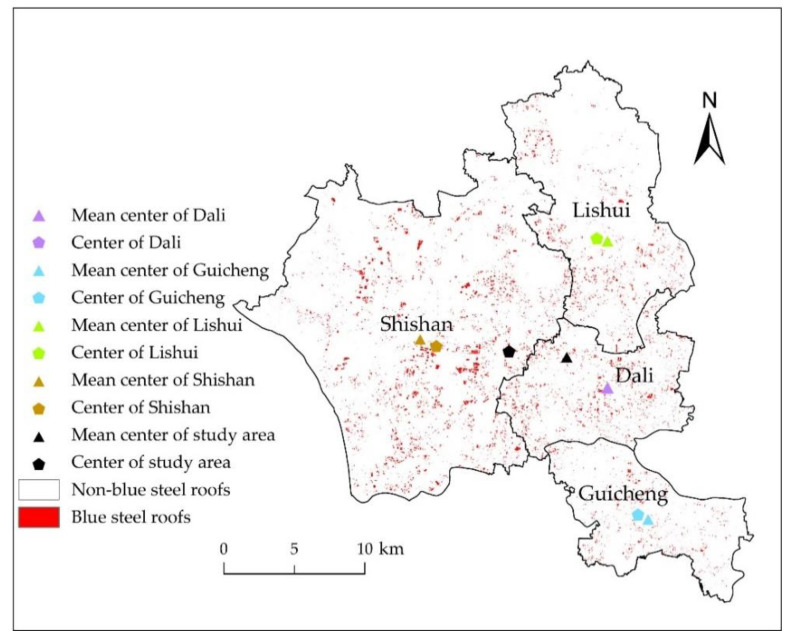
Spatial distribution center and mean center of blue steel roofs.

The mean center is calculated by ArcGIS software according to Equation (7) and the weighted mean center according to Equation (8). Figure 7, the map of the spatial distribution of blue steel roofs and the center reveals that the spatial distribution of the blue steel roofs showed some degree of clustering and uneven distribution at the scale of the study area and that the distribution was concentrated in Shishan. At the town scale, the deviation in the mean center and distribution center was not large. The blue steel roofs within the Guicheng, Dali, and Lishui regions were evenly distributed, and the distribution type of Shishan was generally uniform.

The total area of blue steel roofs was 17.84 km^2^. If the blue steel roofs area was allocated proportionally to each town (i.e., the geographical area of each town accounted for the total geographic area of the research area)—2.27 km^2^ (Guicheng), 2.48 km^2^ (Dali), 4.01 km^2^ (Lishui), and 9.09 km^2^ (Shishan)—then G was 58.76. However, the value of G, when the proportion of the different cites is not accounted for, was 62, indicating that the blue steel roofs distribution in this area was relatively clustered. C of the blue steel roofs in the study area was 0.17, indicating that the distribution of the blue steel roofs was uneven.

G and C of each town were also calculated separately to explore the distribution types of blue steel roofs within towns themselves (Table 4). G of Shishan was 9.36, which was the highest among the four towns. C of Shishan Town was 0.41, corresponding to a generally uniform spatial distribution type. With the exception of Shishan, C was higher than 0.6, and the distribution type was uniform.

### 4.3. Area of Blue Steel Roofs

**Figure 8:**
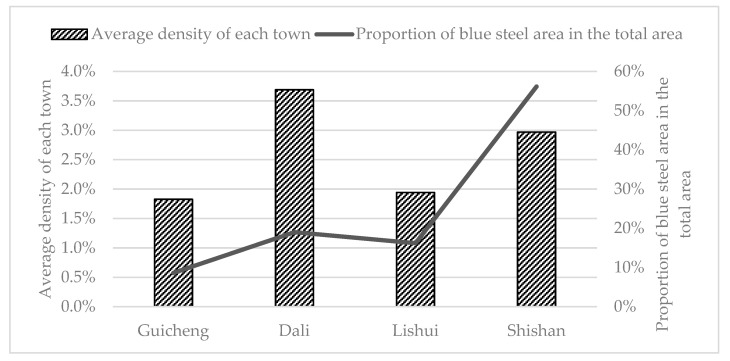
Proportion of blue steel roofs area out of the total area and average density.

The study area included four towns—Shishan, Dali, Lishui, and Guicheng—and the total geographical area was 662.37 km^2^. The town with the largest geographical area (Shishan) was 337.36 km^2^, followed by Lishui (148.82 km^2^) (Figure 8). The town with the smallest geographical area was Guicheng (84.28 km^2^), followed by Dali (91.92 km^2^). Based on the vector statistics in ArcGIS, the town with the largest area of blue steel roofs was Shishan (10.02 km^2^), followed by Dali (3.39 km^2^). The town with the smallest area was Guicheng (1.54 km^2^), followed by Lishui (2.89 km^2^). The proportion of blue steel roofs out of the total area was 8.62% (Guicheng), 16.20% (Lishui), 19.01% (Dali), and 56.16% (Shishan), respectively. If the ratio of the area of blue steel roofs to the total geographical area is assumed to represent the average density of blue steel roofs of Guicheng, Dali, Lishui, and Shishan, then the average density of blue steel roofs in the four towns was 1.83%, 3.68%, 1.94%, and 2.97% respectively. Dali is the town with the highest average density of blue steel roofs, and Guicheng is the town with the lowest average density of blue steel roofs. Thus, Shishan accounts for more than half of the blue steel roofs in the study area, and the other three towns account for less than 50%.

**Figure 9:**
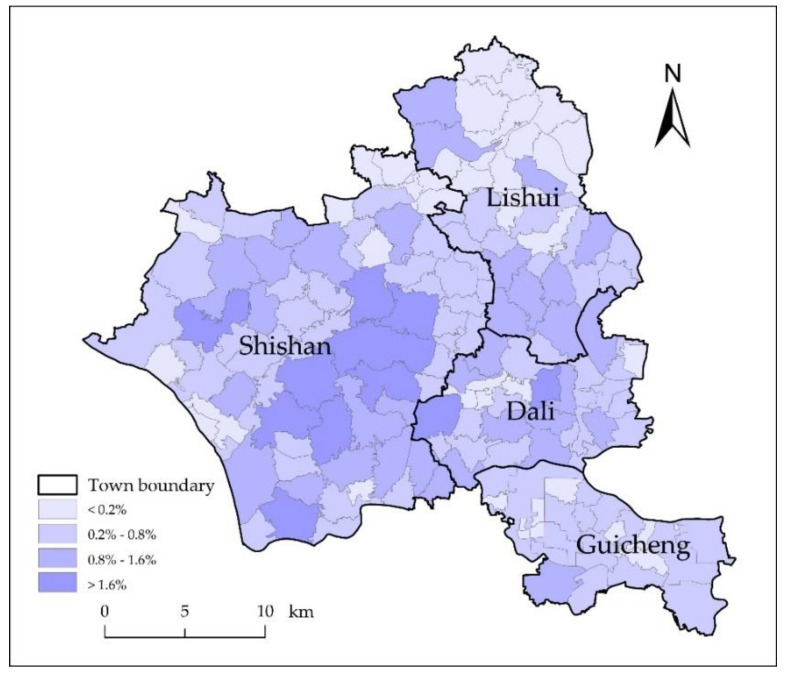
Proportion of blue steel roofs area to total blue steel roofs area.

Calculations of the geographical area and blue steel roofs area of the villages show that Jianxing in Lishui, is the village with the lowest average density of blue steel roofs. The area of blue steel roofs was only 0.0012 km^2^, and the average density was 0.02%. The village with the highest average density of blue steel roofs was Xingxian in Shishan (9.50%). Figure 9 shows the proportion of the blue steel roofs area relative to the total blue steel roofs area. Among areas of blue steel roofs for each village, Shishan had the largest proportion, followed by Dali. Shishan not only had a large blue steel roofs area but also had an average density of blue steel roofs. Among all towns, Shishan had a higher number of large blue steel roofs buildings relative to the other towns.

In addition, the area of blue steel roofs is also closely related to the study of inefficient industrial urban land. In Nanhai, Shunde, and other places, the proportion of urban construction land has exceeded 40% and is only 21% in Hong Kong and 16.4% in Japan’s three metropolitan areas. Therefore, if the construction of urban land is not regulated and managed, available land may be depleted [47]. In 2016, the former Ministry of Land and Resources issued a notice of “guiding opinions on further promoting the redevelopment of urban low utility land (implementation)”. To date, nationwide research and verification of the low efficiency of urban land construction have been conducted on a large scale. For industrial land, detailed identification and evaluation would greatly improve the development and future direction of urban development, as the fundamental purpose is to promote the sustainable use of land resources along with conservation and intensive use [48]. The identification of inefficiently designed industrial land can be made based on considering three aspects: land production efficiency, the utilization rate of industrial land, and the adequacy of social service function [49]. The evaluation indicators can be determined based on the aforementioned ideas relating to low-efficiency industrial land evaluation. Among these indexes, there is a need to make calculations according to the area of the industrial land building, such as the average output intensity of the land, the average tax of the land, and the provision of jobs per unit area. For some heavy industrial plants using steel frame structures, the area of blue steel roofs can reflect the area of industrial land, which is important for conducting preliminary assessments of areas of inefficient industrial land and for regional planning and coordination.

### 4.4. Correlation with Social Economic Data

GVIOADS, RLF, ICECADS, Population, and GVIO were analyzed as potential factors that could explain the distribution of blue steel roofs. The results of the correlation analysis are shown in Table 5.

Population and blue steel roofs area showed a weak positive relationship (r = 0.487, *p* = 0.513). GVIOADS, RLF, and GVIO all showed strong positive correlations with blue steel roofs area (r = 0.988, *p* = 0.012; r = 0.971, *p* = 0.029; and r = 0.985, *p* = 0.015, respectively). ICECADS also was highly and significantly positively correlated (r = 0.995, *p* = 0.005).

With the development of the construction industry, colored steel plates have evolved with the development of steel structures and have gradually replaced the traditional building structures, and have come to be widely used in major industrial buildings [50,51]. In some heavy industries, such as metallurgy, machinery, and automobile manufacturing, where large and medium-sized machine tools and complete sets of equipment are used, plants are generally constructed with a single-layer frame structure to meet the requirements of placing large and heavy equipment in the workshop to produce heavy products [52]. Nowadays, the roofs of these steel-structured industrial plants generally use colored steel panels, such as single-layer colored steel roofs or double-layer colored steel tile on-site composite glass wool roofs [53].

Among correlations between the area of blue steel roofs and potential influencing factors, the correlation between ICECADS and the area was the highest, followed by GVIOADS. Because some heavy industrial plants need to use colored steel roofs with steel frames, the area of blue steel roofs is closely related to ICECADS and GVIOADS. According to the Statistical Yearbook of the Nanhai District in 2016, the GVIO of heavy industry accounted for 66.28% of the GVIO of Nanhai District, and the Industry Energy Consumption of heavy industry accounted for 77.88% of Nanhai District.

**Figure 10:**
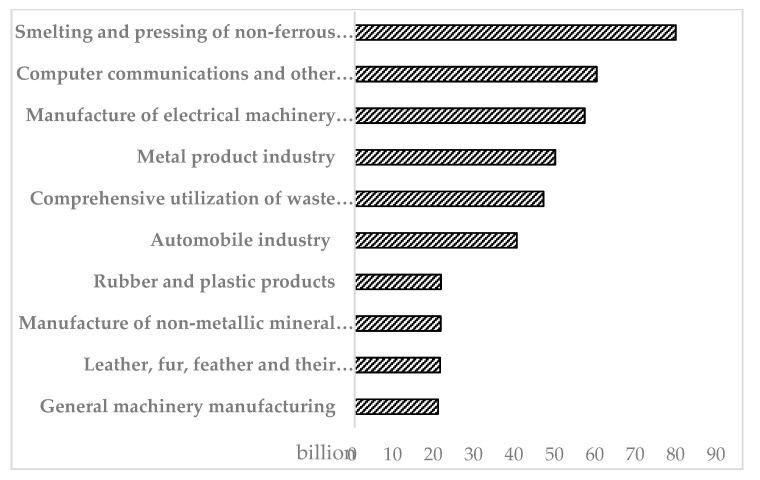
GVIOADS by industry (top 10).

**Figure 11:**
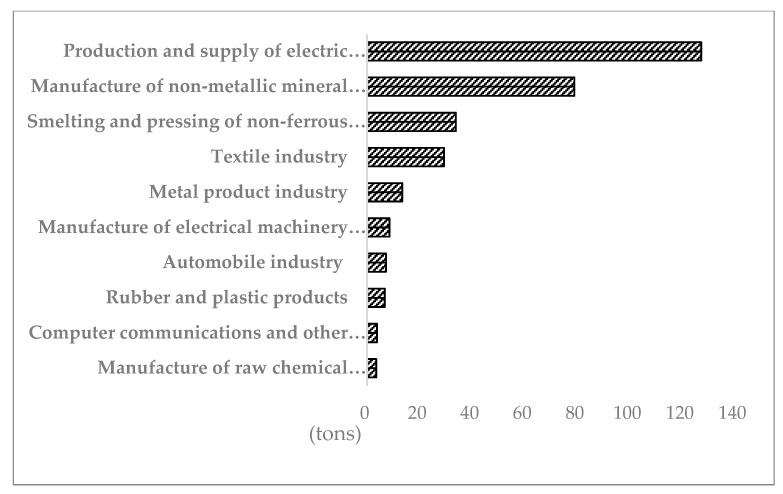
ICECADS by industry (top 10).

Further analysis of industrial enterprises in Nanhai District by industry shows that blue steel roofs may be more likely to be used in factories in industries that contribute more to GVIO and ICECADS. The top 10 (Figure 10) of the GVIOADS by industry accounted for 73.35% of the total value. Some industries may occupy large areas, require large-scale equipment, and use colored steel roofs and steel frame plants. These include the smelting and pressing of nonferrous metals, the manufacture of electrical machinery and equipment, metal product industry, automobile industry, and the manufacture of nonmetallic mineral products. The top 10 of the ICECADS is shown in Figure 11; they accounted for 91.04% of the total value. The industries that might use the colored steel roofs are production and the supply of electric power and heat power, the manufacture of nonmetallic mineral products, production and supply of electric power and heat power, metal product industry, automobile industry, manufacture of electrical machinery and equipment, and manufacture of raw chemical materials and chemical products.

## 5. Discussion

**Figure 12:**
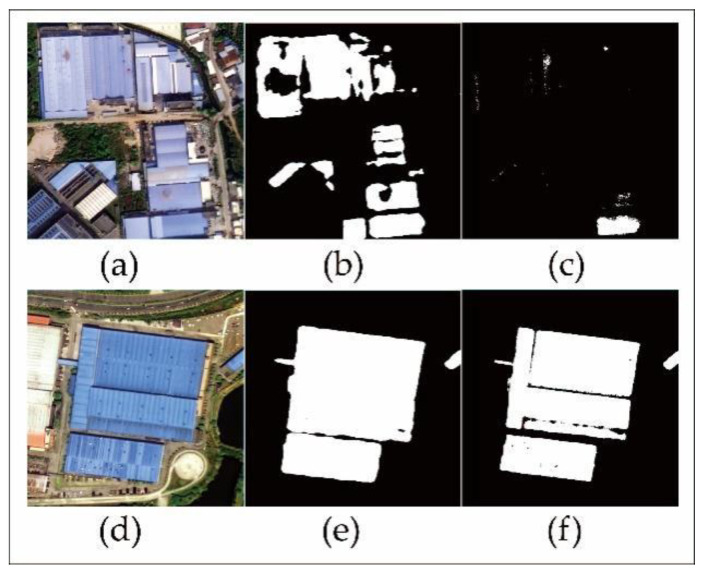
Comparison of the extraction results based on the deep learning method and the maximum likelihood classification method: (**a**,**d**) show the GF-2 images of the second and twelfth regions, respectively; (**b**,**e**) show the extraction results of the deep learning method in the second and twelfth regions, respectively; and (**c**,**f**) show the extraction results of maximum likelihood classification (MLC) in the second and twelfth region, respectively.

To evaluate the performance of the deep learning method, the results were compared with the traditional maximum likelihood classification (MLC) method using the same remote sensing images. The comparison of the extraction results of the two methods is shown in Figure 12. The DeeplabV3+ semantic segmentation model better extracted the boundary frame of the buildings, expressed the contour information of the building overall, and produced a more complete and detailed extraction with fewer misclassifications [54]. When selecting samples for visual interpretation, the DeeplabV3+ method only needed to select blue steel roofs samples; in contrast, MLC needed to select both blue steel roofs and nonblue steel roofs training sample areas.

**Figure 13:**
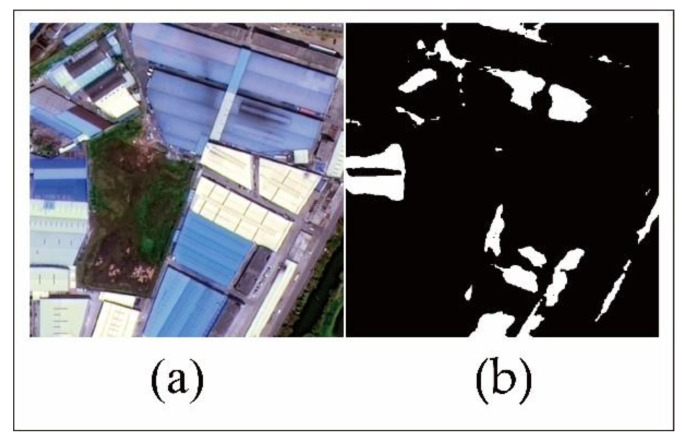
The extraction results and the actual distribution of blue steel roofs in the tenth area: (**a**) is the GF-2 image, and (**b**) is the extraction results.

A comparative analysis of the four evaluation indexes revealed that the 10th region had the lowest values of the 20 regions among the verification samples: precision, 0.43; recall, 0.20; accuracy, 0.92; and *F*1-score, 0.27. Based on other regions with high values, the low detection accuracy in the tenth region can be explained by the dimension of blue steel roofs and the shape, texture, and color of the surrounding buildings. The forecasting results and the distribution of blue steel sheds in the tenth area are shown in Figure 13. The color change is what makes the model unable to distinguish the blue steel roofs from other buildings. Thus, the method used in this study still shows potential room for improvement for reducing the false detection rate.

In future research, further improvement of extraction accuracy could be achieved by adding samples and adjusting model parameters to ameliorate the low accuracy of small-scale targets and color recognition. To improve land-use efficiency and improve spatial quality, it would be useful to continue to use the model to learn more information about other features and extract data on various types of urban land use to elucidate the rate of urban land-use change and regional diversity. The experimental results could also be combined with meteorological data to develop ways of reducing the impact of the heat island effect.

## 6. Conclusions

This study focused on three aspects: the extraction of blue steel roofs from Gaofen-2 remote sensing images, the analysis of the spatial distribution of blue steel roofs, and discussions of the correlation between the area of blue steel roofs and economic factors. The main research results and conclusions are detailed below.

(1) The DeepLabV3+ deep learning model performed well in extracting the blue steel roofs information in Nanhai District (Lishui, Dali, Shishan, and Guicheng) of Foshan City. The overall accuracy was 92%, which is better than the maximum likelihood classification methods.
(2) The distribution of blue steel roofs was not even across the whole study area, indicating regional clustering of the factories.
(3) The blue steel roofs areas were positively correlated with economic factors, such as GVIOADS, RLF, and ICECADS, proving that it might serve as an indicator for inefficient industrial areas in regional planning and its environmental and socio-economic significance.

## Figures and Tables

**Table 1:** Statistical yearbook information for the study area.

Name	Area (km^2^)	RLF	GVIOADS (Billion Yuan)	ICECADS (Tons)	Population	GVIO (Billion Yuan)
Guicheng	1.54	76,425	30.56	62,022	262,646	43.46
Shishan	10.02	198,562	323.23	1,016,390	297,423	341.40
Dali	3.39	113,299	54.63	182,612	263,734	60.08
Lishui	2.89	72,821	84.61	133,177	138,284	90.81

RLF: rural labor force; GVIOADS: gross value of industrial output above designated scale; ICECADS: industrial comprehensive energy consumption above designated scale; GVIO: gross value of industrial output.

**Table 2:** Training Parameters.

Parameter	Value
Base learning rate	0.005
Batch size	4
Weight decay	0.0002
Max iteration times	10,000

**Table 3:** Strength of correlation indicated by different correlation coefficient values.

Correlation Degree	Complete Correlation	High Correlation	Significant Correlation	Low Correlation	Micro Correlation	No Correlation
|R|	1	0.8~1	0.5~0.8	0.3~0.5	0.3~0	0

**Table 4:** Distribution type of blue steel sheds in each town.

Name	Geographic Concentration Index	Uniformity	Distribution Type
Dali	4.37	0.73	Uniform
Guicheng	2.20	0.86	Uniform
Lishui	3.84	0.78	Uniform
Shishan	9.36	0.41	Generally uniform

**Table 5:** Pearson correlation coefficients of several factors potentially explaining the distribution of blue steel roofs.

Factor	Pearson	*p*-Value
GVIOADS	0.988 *	0.012
RLF	0.971 *	0.029
ICECADS	0.995 **	0.005
Population	0.487	0.513
GVIO	0.985 *	0.015

* indicates that the correlation is significant at 0.05; ** indicates that the correlation is significant at 0.01.
